# Prevention of Fractures in Older People with Calcium and Vitamin D

**DOI:** 10.3390/nu2090975

**Published:** 2010-09-16

**Authors:** Caryl A. Nowson

**Affiliations:** School of Exercise and Nutrition Sciences, Deakin University, 221 Burwood Highway, Burwood VIC 3125, Australia; Email: caryl.nowson@deakin.edu.au

**Keywords:** calcium, vitamin D, fracture, meta-analysis

## Abstract

The greatest cause of fracture in older people is osteoporosis which contributes to increased morbidity and mortality in older people. A number of meta-analyses have been performed assessing the effectiveness of calcium supplementation alone, vitamin D supplementation alone and the combined therapy on bone loss and fracture reduction in older people. The results of these meta-analyses indicate that vitamin D supplementation alone is unlikely to reduce fracture risk, calcium supplementation alone has a modest effect in reducing total fracture risk, but compliance with calcium supplements is poor in the long term. The combination of calcium supplementation with vitamin D supplementation, particularly in those at risk of marginal and low vitamin D status reduces total fractures, including hip fractures. Therefore older people would be recommended to consume adequate dietary calcium (>1100 mg/day) together with maintaining adequate vitamin D status (>60 nmol/L 25(OH)D) to reduce risk of fracture. It is a challenge to consume sufficient dietary calcium from dietary sources, but the increasing range of calcium fortified foods could assist in increasing the dietary calcium intake of older people. In addition to the usual dairy based food sources, vitamin D supplements are likely to be required for older people with reduced mobility and access to sunlight.

## 1. Introduction

Lifestyle factors that contribute to the development of osteoporosis occur throughout life, spanning as far as prenatal exposure of the foetus to environmental factors [1]. The adverse outcomes of osteoporosis do not manifest themselves until later life, when incidence of fractures increases. The greatest cause of fracture in older people is osteoporosis which contributes to increased morbidity and mortality in this age group. In women with osteoporosis, the risk of fracture increases from 15% in those aged between 60–64 years up to 71% for women aged >80 years, with a similar age-related increase in men: 1.6% in those aged between 60–64 years up to 19% for men aged >80 years, albeit at a much lower incidence [2]. In Australia, one half of all women and one third of men over 60 will have a fracture due to osteoporosis. Common sites are: hip (16%), spine (46%), wrist (16%), ribs, pelvis and upper arm. Osteoporosis is a major cause of injury, long term disability and death in older Australians: 1/5 of those who fracture a hip will die within 6 months and of the survivors, 1/2 will not be able to walk without assistance and 1/2 half will need full-time nursing care [3].

## 2. Dietary Predictors of Fracture in Older People

As people age, the physiological reserve is often limited and this increasing loss of physiological reserve is called frailty, which in more advanced stages is associated with muscle weakness and increasing disability. Such people often have a slow and unsteady-gait, with an increased risk of falling and sarcopenia and osteopenia which increase susceptibility to fracture [4]. The common set of risk factors for hip fracture have been found to be: low bone mineral density (BMD), postural instability and/or quadriceps weakness, a history of falls, and prior fracture [5].

In older people, age-related bone loss in both sexes is usually in the range of 0.5–1.0% per year with an apparent increasing rate of bone loss at the femoral neck with age [6], which contributes to increased risk of fracture with ageing. There is no cure for osteoporosis, but there is evidence that some lifestyle factors, notably dietary calcium, vitamin D and exercise may reduce this age-related reduction in bone mass. Across most societies, particularly in older people, dietary calcium intake does not match dietary requirements. In the last National Nutrition Survey (NNS), the average intake of dietary calcium for women over the age of 65 years was only 685 mg per day [7]. In Australia the current Recommended Dietary Intake (RDI) (designed to meet the needs of at least 98% of the population) is 1,300 mg/day for women >50 years and men >70 years, with a corresponding Adequate Intake (AI) of 1,100 mg calcium per day) (which meets needs of at least 50% of the population) [8]. Although we have no good population data on the vitamin D adequacy of the population, based on serum vitamin D 25(OH)D measurements, it has been estimated that between 45–75% of older adults (>60 years) are vitamin D insufficient (25(OH)D ≤ 50 nmol/L) [9]. The NNS also indicated that dietary intake does not come close to meeting dietary requirements. As the estimated median vitamin D intake from food is in the range 1.5–2.5 µg/day [10], and the estimated Adequate Intake (AIs) for older people ranges from 400 IU(10 µg)/day for those 50–70 years to 600 IU(15 µg)/day for those >70 years [8].

## 3. Scope of Review

Vitamin D status and calcium homeostasis are inextricably linked in the body and cannot be considered independently, particularly with respect to fracture risk. The aim of this paper is to review the evidence base, from meta-analyses conducted using data from randomized controlled trials addressing the effectiveness of calcium and vitamin D supplementation as individual nutrients or as combined therapies on overall fracture risk. The groups that are most likely to benefit from supplementation will be discussed together with the real world challenges in achieving adequate vitamin D status and sufficient dietary calcium in older people.

## 4. Vitamin D and Risk of Falls

In addition to the well characterized effects of vitamin D on bone, vitamin D is now recognized as being important in maintaining muscle strength and power. Worldwide there is an increase in falls an fractures in winter months, following the seasonal drop in serum 25(OH)D, and this effect is evident even in Australia where ice and snow are not contributing factors [11]. A number of studies have demonstrated that older people with serum 25(OH)D levels <22.5 nmol/L had poorer lower extremity function and higher falling rates compared with those with 25(OH)D ≥ 22.5 nmol/L [12]. Furthermore it has been demonstrated that vitamin D supplementation taken by those in the insufficiency range (25–90 nmol/L) in residential care establishments does reduce the falls rate [13]. This Australian randomized controlled study of vitamin D supplementation equivalent to 1,000 IU (25 µg) vitamin D_2_ (combined with calcium supplementation (600 mg)), demonstrated a 30% reduction in the rate of falling, in the group who took at least 50% of the supplements over a two year period [13]. A recent meta-analysis of vitamin D supplementation and rate of falls, which included eight trials (6 of which included co-administration of a calcium supplement (n = 2,426) found that high dose (7 trials n = 1,921) (700–1,000 IU) reduced falls by 19% [14]. However calcium supplementation was co-administered in 6 of the 7 high dose trials, and none of the 4 low dose trials. Furthermore results from one trial (with different 3 doses of vitamin D, without co-administration of calcium) were overrepresented in this meta-analysis, as this one trial accounted for one of the high dose trials and 3 of the low dose trials [15]. This paper also pooled the results from 15 eligible less stringent studies (n = 17,786) and found that achievement of serum levels 25(OH)D > 60 nmol/L resulted in a 23% reduction in falls [14]. It therefore appears that vitamin D supplementation combined with co-administration of calcium does reduce the rate of falls in older people, but there are insufficient data to make any firm conclusions regarding the effectiveness of vitamin D supplementation on rate of falls without a concurrent increase in dietary calcium. 

## 5. Vitamin D and Fracture

There have been a number meta-analyses that have assessed the impact of vitamin D supplementation on fracture and the results differ depending on the selection of studies included. In a meta-analysis that assessed the effect of calcium combined with vitamin D in 8 studies (n = 52,625), there was a 13% reduction in total fractures [RR = 0.87 (0.77, 0.97)] [16]. One other meta-analysis attempted to define the minimum dose to achieve a reduction in fracture rate by splitting studies into low (<400 IU/day) and high doses of vitamin D (>400 IU/day) in those >65 years [14]. This analysis indicated, on doses more than 400IU, there was a 20% reduction in non vertebral fractures (n = 33,265) (9 trials) which was characterised by a 29% reduction in community-dwelling older people and a 15% reduction in institutionalized older [14]. However it should be noted that the Women’s Health study was classified in the high dose trials, although the supplement used was only 400 IU/day [17] on the basis that the background dietary intake of US is relatively high due to fortification. Although the authors indicate that reduction in non-vertebral fractures with the higher dose >400 IU was independent of additional calcium supplementation [14], most studies included in this meta-analysis used combined vitamin D and calcium. Of the 12 trials included (total n = 42,279), 7 used vitamin D combined Ca with calcium. The recent meta-analysis performed on a smaller number of trials, but at the more powerful patient level of 68,500 patients from 7 major vitamin D fracture trials in US and Europe [18] did not confirm a dose response of vitamin D. This study found that the effect of vitamin D supplementation was independent of age, gender, or previous fracture. The authors concluded that vitamin D given alone in doses of 400–800 IU (10–20 µg) was not effective in preventing fractures, but that combined calcium and vitamin D reduced hip fractures and total fractures, and probably vertebral fractures.

Finally, the most recent Cochrane review addressing the effectiveness of vitamin D with or without calcium supplements on the risk of fracture, clearly indicated that vitamin D alone is not effective in preventing hip fracture (nine trials, 24,749 participants, RR 1.15, 95% CI 0.99 to 1.33), vertebral fractures or any new fracture (ten trials, 25,016 participants, RR 1.01, 95% CI 0.93 to 1.09) [19]. 

It is important, to acknowledge that large doses of vitamin D can lead to adverse health outcomes. Results from a double-blind, randomized controlled trial of 2256 community-dwelling women, aged 70 years or older who were at greater risk of fracture, indicated that a single annual dose of 500,000 IU of cholecalciferol increased fractures by 26%, with more fractures evident within the first 3 months [20]. The results of this trial clearly indicate that there are adverse health effects with such large doses.

## 6. Calcium and Fracture

A meta-analysis was conducted to simply answer the question should calcium supplementation be recommended for older people and is there a significant health benefit in terms of reducing bone loss and reducing overall fracture risk in older people (>50 years) [16]? This meta-analysis included randomized controlled trials that administered either calcium alone or calcium combined with vitamin D. The results clearly indicated a beneficial effect of calcium supplementation in reducing bone loss in a total sample of 63,897 older people of which 92% were women and the mean age was 68 years. Calcium supplementation reduced bone loss at the hip 0.54% [(95% CI 0.35, 0.73) p < 0.0001] and at the spine by 1.19% [(95% CI 0.76, 1.61) p < 0.0001]. When assessing the effect of calcium alone or calcium combined with vitamin D on total fractures, there was a clear benefit across 17 studies: (n = 52,625), where treatment reduced total fractures by 12% and where 63 patients needed to be treated 3.5 years with calcium with or without vitamin D to prevent one fracture. This magnitude of a health benefit compares favourably to other more expensive therapies for cardiovascular disease (CVD) where for statins 40 need to be treated for 5 years to prevent 1 CVD event. When Tang *et al.* [16] split the studies into those supplementing with calcium alone and those supplementing with calcium combined with vitamin D, and assessed the effect on the rate of fracture there was no significant difference, but there was a trend for the combined therapy to have a greater effect. It should be noted that all the results of all the trials were in direction favourable for an effect, but the sample size was considerable less for those trials using the combined therapy (n = 6,517) compared to Ca alone (n = 52,625). Calcium supplementation alone reduced total fractures by 10% [RR = 0.90 (0.80, 1.00)], and combined therapy by 13% [RR = 0.87 (0.77, 0.97)]. The more recent DIPART meta-analysis performed at patient level also demonstrated that calcium supplementation combined with vitamin D supplementation reduced all fractures by a comparable level 8% [RR = 0.92 (95% CI, 0.86, 0.99)]. 

### Hip Fractures

As hip fractures have the greatest impact in reducing quality of life and increased mortality, there is some controversy regarding the effectiveness of calcium supplementation in reducing hip fracture with one randomized controlled trial indicating an increased risk of hip fracture [21]. It is therefore relevant to assess the meta-analysis relating to hip fracture. The recent DIPART meta-analysis performed at patient level with 68,500 patients, indicated a 16% reduction in hip fracture, which did not quite reach significance [RR = 0.85 (95% CI, 0.70, 1.01)] for calcium combined with vitamin D [18]. One meta-analysis assessing the impact of calcium supplementation specifically on hip fractures, which included 4 RCTs, with calcium supplementation between 800–1,200 mg/day, found a pooled relative risk was 1.64 (1.20, 2.64), however the total sample size was relatively small (n = 6,504) compared to other similar analysis [22]. Another meta-analysis that assessed the effect of vitamin D supplementation alone on hip fracture in 5 studies (total n = 9,083), found no effect: [RR = 1.10 (0.89, 1.36)] [23]. Importantly when assessing the effect of vitamin D supplementation combined with co-administration of calcium in 6 trials, with a much greater total sample size of 45,509, there was an 18% reduction in hip fracture [RR = 0.82 (0.71, 0.94)]. There are difficulties in interpreting results from the Women’s Health Initiative (WHI) study as the mean baseline intake of calcium and vitamin D was relatively high and those on estrogen replacement therapy were included [17]. When a meta-analysis was performed excluding the WHI (n = 36,282) [17] there was a indication of a greater protective effective of calcium supplementation reducing the rate of hip fracture from 18% to 21% (RR 0.79 (0.64, 0.97)) (n = 9,227) [23]. Comparison of combined vitamin D and calcium vs. vitamin D alone for hip fracture indicated that the combination therapy reduced the risk of hip fracture by 25% [RR = 0.75 (0.58, 0.96)] and the risk of all non-vertebral fracture by 12% [RR = 0.88 (0.78, 0.99). This analysis also demonstrated, on a higher dose of vitamin D there was 18% reduction in hip fracture (5 trials) (n = 31,872), which included a 21% reduction in community-dwelling older people and a greater effect in institutionalized older people of 28%. One other meta-analysis which assessed the impact of vitamin D combined with calcium compared to placebo in elderly women, found a reduced rate of hip fracture [OR = 0.70 (0.53, 0.90)] [24]. Finally, the most recent Cochrane review, consistent with the previous meta-analyses, indicated that calcium co-administered with vitamin D reduced hip fractures by 26% (eight trials, 46,658 participants: [RR = 0.84 (0.73, 0.96)] with a non-significant trend for a greater reduction in those in residential care [19]. In summary, most meta-analyses have not confirmed the adverse effect of calcium supplementation (when combined with vitamin supplementation) on hip fractures, with most demonstrating a significant reduction in fracture risk. 

## 7. Groups that Would Benefit Most from Calcium Supplementation

The meta analysis by Tang *et al.* [16] indicated that the groups that gained most benefit from calcium supplementation in terms of fracture, were those who were older: institutionalised, who had a low baseline calcium intake (<700 mg/day), were prescribed a higher dose calcium supplement, and had low serum 25(OH)D < 25 nmol/L levels (P = 0.06) (Table 1).

**Table 1:** Population sub-groups reduction in total fracture risk with calcium supplementation: meta-analysis. Reproduced from [16].

	n	% Risk Reduction	Interaction
50–70 years	36,640	3%	P = 0.003
70–80 years	12,481	11%	
>80 years	3,504	24%	
Serum 25(OH)D < 25 mmol/L	10,144	14%	P = 0.06
Serum 25(OH)D ≥ 25 mmol/L	39,167	6%	
Ca Supp. < 1200mg/d	47,359	6%	P = 0.006
Ca Supp. ≥ 1200mg/d	5,266	20%	
Dietary calcium < 700 mg/d	7272	20%	P = 0.008
Dietary calcium ≥ 700 mg/d	45,241	5%	
Community dwelling	49,233	6%	P = 0.003
Residential care	3,392	24%	
<1% change Bone Mineral Density	38,212	4%	P = 0.007
≥1% change Bone Mineral Density	5,621	20%	

Meta-regression analysis also confirmed the greater effectiveness in those who were older, had a lower body weight, and had a greater baseline risk (e.g., living institutional settings or history of previous fracture).

A smaller reduction in fracture risk was associated with increasing trial duration, and this could be largely attributed to reduced compliance with calcium supplementation over time. Compliance with calcium supplementation was a key predictor of fracture risk. The overall reduction in total fracture risk was 12%, but when the 8 trials, (with an average reported of compliance >80%) were assessed separately there was a doubling of the risk reduction to 25%: [RR = 0.76 (0.67, 0.86)]. The treatment effect was greater in those trials with higher compliance, although the vast majority of individuals participating in the trials recorded compliance with calcium supplementation less than 59% (Table 2). Although an increased risk of cardiovascular disease has been reported with calcium supplementation [25] this not a consistent finding and calcium supplements would be recommended when older people are much less than the recommended intake of calcium from food sources.

**Table 2:** Population sub-groups reduction in total fracture risk with calcium supplementation: meta-analysis. Reproduced from [16].

% Compliance	subtotal n (% total sample)	% reduction in fracture risk
80%	4,508 (9%)	24%
60–69%	3,511 (7%)	8% (ns)
50–59%	44,494 (85%)	4% (ns)

Compliance with vitamin D supplements are thought to be better, as there are generally few adverse side effects. Additionally daily supplementation is not necessary as it is a fat soluble vitamin stored in the body. Regrettably larger dose preparations, that allow for weekly or monthly supplementation regimes, which could significantly reduce the burden of daily supplementation, are not generally available in Australia.

Unfortunately, an adequate amount of calcium is required on a daily basis and long term compliance with calcium supplementation is poor, particularly with calcium carbonate that can cause gastrointestinal side effects. The reality is that many women after the age of 50 would need to take daily calcium supplements for more than 30 years, to meet their calcium requirement as few consume the recommended amounts of dietary calcium on a daily basis. In older people, when energy intakes are lower, achieving an adequate calcium intake (AI) (calcium >1,100 mg/day) is difficult to achieve from dietary sources and requires consumption of at least 3 glasses of milk per day or the equivalent dairy food, e.g., cheese or yoghurt or a equivalent amount of calcium fortified soy products. It is, however, now possible to achieve close to the adequate intake of calcium with a reduced volume of dairy or equivalent products (only 1.5 glasses of milk per day), by using a selection of calcium fortified products now available, e.g., calcium fortified milk (ranging from 350–500 mg calcium per 250 mL serve) (which equates to 1.2–2 times that of standard milk) calcium fortified orange juice (5× higher), bread (10× higher), breakfast cereals (100× higher) (Table 3). 

**Table 3:** Achieving Adequate Intake (AI) for those ≥70 years, for calcium intake utilising a range of calcium fortified products.

Calcium fortified food products	Serving size (g/mLs)	Calcium (mg)
2 slices bread, e.g., UP^®^	60 g	200
1 glass milk, e.g., Anlene^®^, Pura Boost^®^	250 mL	500
½ glass milk on cereal	125 mL	250
1 breakfast cereal, e.g., Special K^®^	30g	200
1 orange juice, e.g., Berri Multi V^®^	250 mL	100
**Total calcium (mg/day)**		1250

## 8. Conclusions

The large volume of data from well controlled randomized controlled trials confirm the effectiveness of calcium supplementation combined with vitamin D supplementation in reducing fracture risk in older people. There is significant evidence indicating that this combined therapy is effective in reducing the rate of falls in older people (within the range of 13–28% depending on dosage), which in addition to the positive effect on bone, contributes to the reduction in fracture risk. Given the ubiquitous distribution of vitamin D receptors throughout the body in every nucleated cell, and the increasing physiological link between vitamin D and muscle metabolism it is likely that vitamin D contributes to positive effects seen with the combined supplementation of calcium and vitamin D in reducing falls. It has previously been established that high intake of calcium appears to have role in maintaining adequate vitamin D status. Dietary calcium and phosphate intake influence vitamin D metabolism and it has been shown in rats that the rate of inactivation of vitamin D in the liver is increased by calcium deprivation [26]. It is clear from the studies to date that vitamin D supplementation alone, without sufficient dietary calcium, does not reduce fracture risk. Supplementation with dietary calcium does have a modest effect in reducing overall fracture risk (~10%), but given the positive effects of vitamin D on falls and fractures and probably other health parameters, health recommendations for older people should include advice to ensure adequate vitamin D status as well as adequate dietary calcium. This combined therapy results in somewhere between a 14–23% reduction in non-vertebral fractures. The effectiveness of combined calcium and vitamin D supplementation is more effective in those who are on lower intakes of dietary calcium and are at risk of having marginal and low vitamin D status (<60–80 nmol/L serum 25(OH)D). 

Few older women and men meet the recommended level of dietary calcium through dietary sources, but providing additional calcium in the form of supplementation to those who are already consuming adequate amounts will not result in additional health benefits and assessment of dietary calcium intake would be warranted before recommending supplements. Some older people may be able to consume sufficient calcium through careful dietary planning and achieving adequate dietary calcium through dietary means, would be preferable to supplements as foods contain a range of other nutrients. In contrast adequate vitamin D cannot readily be achieved through dietary sources and the major source is produced by exposure of the skin to ultraviolet light. For many older people with reduced mobility, safe sunlight exposure is difficult to achieve and vitamin D supplements are necessary. Obviously the effectiveness of any lifestyle or supplement regime depends on the level of compliance. Whilst compliance with vitamin D supplementation appears to be less of an issue and does not necessarily have to be consumed daily, compliance with daily calcium supplementation continues to be problematic. Alternate forms of calcium supplements (instead of calcium carbonate) such as citrate may be more acceptable to some on a daily basis although more expensive. There is now the potential for the increasing range of calcium boosted foods to assist older people to maintain higher daily intakes of calcium and reduce the rate of fractures in the community, although milk and calcium containing dairy products will still provide the bulk of dietary calcium.
